# Analysis of Antibody and Cytokine Markers for Leprosy Nerve Damage and Reactions in the INFIR Cohort in India

**DOI:** 10.1371/journal.pntd.0000977

**Published:** 2011-03-08

**Authors:** Rupendra Jadhav, Lavanya Suneetha, Ravindra Kamble, Vidyagouri Shinde, Karuna Devi, Meher Vani Chaduvula, Renuka Raju, Sujai Suneetha, Peter G. Nicholls, Wim H. van Brakel, Diana N. J. Lockwood

**Affiliations:** 1 Stanley Brown Laboratories, The Leprosy Mission, Shahdara, New Delhi, India; 2 Blue Peter Research Centre, Hyderabad, India; 3 Blue Peter Research Centre, LEPRA India, Hyderabad, India; 4 School of Health Sciences, University of Southampton, Southampton, United Kingdom; 5 Leprosy Unit, Royal Tropical Institute, Amsterdam, The Netherlands; 6 London School of Hygiene & Tropical Medicine, London, United Kingdom; University of Tennessee, United States of America

## Abstract

**Background:**

The ILEP Nerve Function Impairment in Reaction (INFIR) is a cohort study designed to identify predictors of reactions and nerve function impairment (NFI) in leprosy.

**Aim of the Study:**

Antibodies to mycobacteria, nerve components and serum cytokine were measured as potential markers for their possible association with reactions and NFI.

**Patients and Methods:**

303 newly diagnosed leprosy patients from two centres in North India were enrolled. Antibodies to PGL-1, LAM (IgG1 and IgG3), ceramide, S100 and TNFα levels were measured using ELISA techniques.

**Results:**

S-100, PGL IgG and IgM antibody levels were lowest in patients with BT leprosy and highest in patients with lepromatous leprosy. LAM IgG1 and LAM IgG3 antibody levels were highest in patients with BL leprosy. Ceramide antibody levels were not correlated with type of leprosy. Levels of all the antibodies tested and TNF α were lowest in patients with only skin reaction. PGL IgM antibody levels were elevated in patients with skin reactions and NFI. Old sensory NFI is associated with significant elevation of PGL IgG, LAM IgG and S100 antibody levels.

**Conclusion:**

These results reveal that the antibody response to mycobacterial antigens, nerve antigens and cytokines are in a dynamic flux and could collectively contribute to NFI in leprosy. The association of multiple markers with old NFI may indicate the contribution of different pathological processes.

## Author Summary

Leprosy is one of the oldest known diseases. In spite of the established fact that it is least infectious and a completely curable disease, the social stigma associated with it still lingers in many countries and remains a major obstacle to self reporting and early treatment. The nerve damage that occurs in leprosy is the most serious aspect of this disease as nerve damage leads to progressive impairment and disability. It is important to identify markers of nerve damage so that preventive measures can be taken. This prospective cohort study was designed to look at the potential association of some serological markers with reactions and nerve function impairment. Three hundred and three newly diagnosed patients from north India were recruited for this study. The study attempts to reflect a model of nerve damage initiated by mycobacterial antigens and maintained by ongoing inflammation through cytokines such as Tumour Necrosis Factor alpha and perhaps extended by antibodies against nerve components.

## Introduction

Leprosy is a chronic granulomatous disease affecting skin and nerve. There is a range of clinical and immunological responses to infection with *Mycobacterium leprae* and the disease manifests as a spectrum. At the tuberculoid end of the spectrum there is a well developed immune response and mycobacteria are eliminated with a granulomatous response in skin and nerve which may produce severe destruction of peripheral nerves[1]. At the lepromatous end of the spectrum there is little cell mediated immunity and mycobacteria proliferate in skin and nerves and macrophages infiltrate skin and nerve but with no organised response. Most patients have one of the borderline types of disease in which some mycobacteria are present with a lymphocytic and macrophage infiltration of skin and nerve. Mycobacterial antigens are presented to the immune system and initiate a T cell response with macrophage activation and the production of pro-inflammatory cytokines. This inflammation in peripheral nerves produces local destruction of nerve structures, with subsequent loss of nerve function, which puts patients at risk of developing impairments. The pathogenesis of leprosy reactions and nerve damage involves either cell-mediated immunity at sites of localisation of mycobacteria (reversal reaction) [2] or immune-complex syndrome due to precipitation of antigen and antibody complexes in tissue spaces and in blood and lymphatic vessels (ENL) [3]. Identifying patients who are at risk of developing nerve damage is therefore a key challenge in leprosy. Various cohort studies have identified clinical risk factors for the development of nerve damage. Studies in Bangladesh [4], Ethiopia [5] and Thailand [6] have shown that multibacillary leprosy (MB), increasing age and the presence of nerve damage at the time of diagnosis are risk factors for the development of further nerve damage. However, few studies have looked at laboratory parameters as risk factors.

Phenolic glycolipid (PGL-1) is a *M.leprae* specific antigen and 90% of lepromatous, but only 50% of tuberculoid patients have antibodies to PGL-1 in proportion to their mycobacterial load [7]. A study in Nepal showed that seropositivity for PGL-1 IgG antibodies when anti-leprosy treatment is started was a significant risk factor for the development of Type 1 reactions (T1R) [8]. However this finding has not been confirmed in other small studies in Nepal [9] and Brazil [10]. We hypothesised that raised PGL-1 levels would be a risk factor for developing reactions and nerve damage as well as being correlated with bacterial load.

Lipoarabinomannan (LAM) is a polysaccharide antigen present in *M. leprae* and is involved in initiating a specific humoral response in leprosy patients [11]. As a B cell immunogen it might have a role in part of the pathogenesis of nerve damage. LAM antigen persists in the body after the completion of antibacterial treatment and has been shown to be associated with leprosy reactions in skin and nerve biopsies [2]. One study has shown an association between raised LAM antibody levels and the occurrence of T1R [12]. LAM was considered as a potential candidate antigen in the pathogenesis of leprosy reactions, especially the late reversal reactions because it may persist in skin and nerve lesions [2]. We hypothesised that LAM antibody levels would be increased in patients with a high bacterial load and in patients with reactions.

Humoral responses against nerve antigens are pathogenic in some peripheral and sensory peripheral neuropathies [13] and may also have a role in leprosy nerve damage. Ceramide and other sphingolipids are recognised as lipid mediators of the immune response and high concentrations of auto-antibodies to neural proteins and lipid antigens have been demonstrated in leprosy patients [14],[15], [16]. It has been reported that leprosy patients across the Ridley-Jopling spectrum show antibodies against ceramide in their sera [17], [18].

S100 is a specific nerve tissue protein. Narayan et al looked for S100 antigen levels in serum and found that 87.5% of the patients had elevated S100 antigen levels. The studies of Vemuri et al [18], Narayan et al [17] and Eustis-Turf et al [14], although important in suggesting a pathogenic role for antibodies in the development of nerve damage, all lack information on the neurological status of the patients thus making it impossible to confirm the association with clinical nerve damage.

Tumour necrosis factor α (TNFα) is a pro-inflammatory cytokine that is particularly implicated in the pathogenesis of mycobacterial infections. Previous studies have shown that TNFα is produced in skin and nerve during leprosy T1R [19]. Raised serum TNFα levels have been reported in Erythema Nodosum Leprosum (ENL) reactions and one could hypothesise that TNFα might leak from sites of inflammation into the circulation and so be a marker of acute leprosy related inflammation. Serum TNFα levels are found to be high in LL and low/undetectable in BT-TT patients [20].

The INFIR cohort comprised 303 newly diagnosed patients with MB in North India who were recruited to a study looking for risk factors for nerve damage and reactions in leprosy patients [21]. After recruitment they were assessed monthly for a year and then every two months until 24 months after diagnosis. This study design enabled us to correlate serological and clinical findings. At recruitment, patients were asked about the presence of new nerve damage which was defined as nerve damage occurring within the last six months. This then allowed us to look at pathological associations of recent or long-term nerve damage. We were therefore able to test the following hypotheses:

1. PGL-1 antibody levels would correlate with bacterial load and be a risk factor for the presence of reaction and nerve damage.
2. LAM antibodies would be elevated in patients with reactions and nerve damage.
3. TNFα levels would be high in patients with T1R and nerve damage since these are acute inflammatory events with local production of TNFα.
4. Anti-ceramide and S100 levels would be increased in patients with new nerve damage and neuritis but not patients with only skin reactions.

Here we report the findings from the baseline when patients were recruited into the study.

## Materials and Methods

### Ethical considerations

No financial incentives were given to participants. However, travel expenses were refunded on occasion and, where relevant, lost earnings of daily labourers compensated. The study adhered to the International Ethical Guidelines for Biomedical Research Involving Human Subjects (CIOMS/WHO, 1993). Permission for the study was obtained from the Indian Council of Medical Research and ethical approval was given by the Research Ethics Committee of the Central JALMA Institute for Leprosy in Agra. This included permission for the skin and nerve biopsies. Written consent was obtained from individual subjects before inclusion in the study, using a standard consent form.

### Design

This was a cohort study of 303 newly registered MB patients. The patients were followed up monthly for one year and every second month during the second year.

### Location

Recruitment of subjects took place in The Leprosy Mission (TLM) hospitals in Naini and Faizabad, specialist leprosy referral centres in Uttar Pradesh, North India. The immunological and histopathological investigations were carried out at the LEPRA Society Blue Peter Research Centre in Hyderabad, Andhra Pradesh and at the TLM Stanley Brown Laboratories formerly located in Miraj, Maharashtra. Normal control sera were obtained from the Department of Transfusion Medicine, Nizam's Institute of Medical Sciences (NIMS), Hyderabad and Miraj Medical Centre (Wanless Hospital), Miraj.

### Study population

The study population comprised newly registered MB patients requiring a full course of Multi Drug Therapy. A detailed description of the study design has already been published [22].

### Definitions of reactions and nerve function impairment

Nerve function assessment: Nerve function impairment (NFI) was present when a patient had either or both of motor or sensory loss which were assessed by voluntary muscle testing and testing with Semmes –Weinstein monofilaments as described previously [21]. NFI was categorised as old when signs and symptoms had been present for more than six months and new when signs and symptoms had been present for six months or less.

Type 1 or Reversal Reaction (T1R): T1R was diagnosed when a patient had erythema and oedema of skin lesions. This may have been accompanied by neuritis and oedema of the hands, feet and face.

A patient could have a skin reaction only, or a nerve reaction, or a skin and nerve reaction.

Erythema Nodosum Leprosum (ENL): ENL was diagnosed when a patient had crops of tender subcutaneous skin lesions. There may have been accompanying neuritis, iritis, arthritis, orchitis, dactylitis, lymphadenopathy, oedema and fever.

### Database

All the data obtained in the study, including the clinical, neurophysiological, serological and histopathological data, were entered on computer locally and subsequently merged into a single Microsoft Access database. Further details of the study plans, methods, definitions, documentation and the status of the cohort at baseline have been published [21].

### Materials

S100 protein, Anti-human IgG Peroxidase conjugate, Monoclonal Anti-human IgG1, Anti-human IgG3, Anti-mouse IgG (gamma chain specific) Ceramide, Anti-human IgM (peroxidase conjugated), Avidin peroxidase, Orthophenyl Diamine (OPD) tablet, Tween 20, Carbonate-bicarbonate buffer capsules, Phosphate citrate buffer tablets and bovine serum albumin (BSA) were obtained from Sigma Aldrich, USA. ManLAM and disaccharide conjugate of PGL was kindly provided by Prof. Patrick Brennan, Colorado State University. Monoclonal antibodies to TNFα (capture antibody), secondary antibody (detection antibody) and recombinant TNFα standard were obtained from R & D Systems, London. Chemicals for phosphate buffer were obtained from Merck, the Immulon-2B ELISA plates from Thermo Labsystems, Finland and the plate sealers from Linbro, ICN, USA.

### Laboratory methods

Serum samples from the patients at the time of recruitment (baseline samples) were tested for various parameters. The different serological parameters tested using ELISA technique were: antibodies to the *M.leprae* – PGL and LAM; antibodies against nerve components S100 and ceramide and the proinflammatory cytokine TNFα.

ELISA for antibodies against S100,PGL-I, LAM and ceramide: Antigens against which the antibodies were to be tested were dissolved in suitable solvent like deionised water (S-100 and PGL-I), or 70% methanol in PBS (ManLAM) or PBS (ceramide). ELISAs were carried out as follows: briefly 96well microtitre plates (Dynatech) were coated with the antigen at a concentration of 0.1 µg/well in 0.05 M carbonate-bicarbonate buffer pH 9.6 by incubating overnight at 37 °C (for S-100, PGL-I and LAM). For anticeramide the antigen in PBS was sonicated prior to coating to obtain uniform suspension. The plates were washed 4 times (4×) in phosphate buffered saline with Tween 20 (PBS-T) and then blocked with 2%BSA in PBS for 1½ hr at 37 °C in a humidified chamber. After washing 4× with PBS-T, test sera (1∶100 dilution) were added (100 µl/well) in triplicate. Standard pooled sera (sera showing high antibody titre against the antigen to be tested) were used for the S-100, PGL-I and LAM ELISAs. Twofold dilutions were used ( 1∶25 to 1∶800 for S-100 and LAM ELISAs, 1∶100 to 1∶3200 for PGL-I IgG and 1∶200 to 1∶6400 for PGL-I IgM).The dilutions were assigned arbitrary units. The lowest dilution of the sera was assigned 200 AU whereas subsequent dilutions were assigned AU values accordingly e.g. 1∶100 dilution for PGL-I IgG was assigned 200 AU whereas 1∶200 dilution was assigned 100 AU and so on. The O.D. values of different dilutions of the standard pooled sera were then plotted against the assigned AU values to get a standard graph. Thus using the same standard pooled sera we could prepare a standard graph to test the antibody levels for all the samples tested so that we could compare the results. Plates were incubated at 37 °C for 1½ hr in a humidified chamber and then washed 4× with PBS-T. For S-100, PGL-I IgG and ceramide ELISAs 100 µl of anti-human IgG- horse radish peroxidase (HRPO) was added to the wells whereas for PGL-I IgM ELISA anti-human IgM-HRPO was used. Plates were incubated at 37 °C for 1½ hr. For LAM IgG1 and IgG3 antibody ELISA the procedure used was as described earlier by Beuria et al [12] i.e. after the steps of addition of test / standard sera and plate washing, and prior to addition of enzyme conjugated antibody, 100 µl of mouse anti-human IgG1or mouse anti-human IgG3 monoclonal antibody (1∶3000 and 1∶2000 diluted respectively) was added to the plate and incubated at 37C for 1½ hr in a humidified chamber and then washed 4× with PBS-T. Then 100 µl goat anti-mouse IgG-HRPO conjugate was added at dilution of 1∶3000 for LAM-IgG1 ELISA or 1∶2000 for LAM-IgG3 ELISA. Plate was incubated at 37 °C for 1½ hr in a humidified chamber and then washed 4× with PBS-T.

After washing, OPD substrate (in phosphate citrate buffer with perborate pH 5.0) at a final concentration of either 0.4 mg/ml (S-100, PGL-I) or 1 mg/ml (LAM-IgG1 and IgG3) was added. For anti-ceramide antibody ELISA OPD substrate (1 mg/ml) in phosphate citrate buffer containing 0.06% H_2_O_2_ was added. The reaction was stopped with 3N HCl (for S-100, LAM and PGL-I) or 3N H_2_SO_4_ (for anticeramide) after incubating at 37 °C for 15 minutes. The optical density (OD) was measured using a dual filter at the wavelength of 490/630 nm (Dynatech).

Estimation of serum TNFα: Sandwich ELISA development kit from R&D was used to detect serum TNFα. Briefly, the monoclonal antibodies to TNFα were coated onto the ELISA plate (Immulon-2B) at a concentration of 300 ng/well and incubated overnight at room temperature (26 °C). The next day, the plate was blocked in 2% BSA and incubated with 200 µl of test sera for 2 hr which are then detected by biotinylated secondary antibodies. The plate was then treated with Avidin peroxidase diluted according to manufacturers' instructions and developed by adding substrate OPD at a concentration of 1 mg/ml in Phosphate Citrate buffer pH 5.0 containing 0.06% H_2_0_2_ for 15 minutes in dark. The reaction was terminated with 50 µl of 3N H_2_SO_4_ and the colour was read at 490 nm. Every step until substrate addition was followed by five washing cycles programmed in Biorad plate washer with PBS pH 7.4 containing 0.1% BSA and 0.05% Tween 20. For TNFα, OD values were entered into the database. For the full data set TNF concentrations (pg/ml) were then computed by applying the manufacturer's standard transformation based on a straight line relationship between the logarithm of the inverse OD and the logarithm of the inverse concentration.

### Analysis of results

Statistical analysis was performed using Stata. The significance of association between outcome and predictor variables was tested using chi-squared tests or Fisher's exact test as appropriate. Differences between means or medians were tested with Student's t test or the Kruskal-Wallis test.

## Results

One hundred and fifteen patients had a reaction or new NFI at baseline. Table 1 gives the details of the nerve impairment found at baseline. One hundred and forty three patients (47.2%) had NFI at baseline. 67 (21.1%) had new impairment detected by monofilament or VMT. Old sensory nerve damage was the commonest impairment, being detected in 104 (34.3%) patients and new sensory damage in 50 (16.5%) patients. Motor damage was detected less often, with 26 (8.6%) patients having old and 33 (10.9%) new motor nerve damage. Fifty four (17.8%) had a T1R. One hundred and ninety three patients of the whole cohort (303) were slit skin smear negative for leprosy bacilli at diagnosis. Of this subgroup 60 had a reaction or NFI at baseline with 22 having a T1R, 39 new NFI and 15 other neuritis. Sera taken at time of diagnosis were available for all patients recruited to the study. This was tested for all seven serological markers. Since the focus of this study was to identify associations with pathological processes and nerve damage the findings are presented first by Ridley-Jopling classification and then by type of reaction and nerve damage.

**Table 1:** Reactions and nerve damage status of 303 subjects recruited into study.

Total number recruited	303
In reaction (all forms)	115 (38.0%)
Not in reaction	188 (62.0%)
**Reaction status (overlapping groups)**	
Type 1 reaction	54 (17.8%)
Type 2 reaction	5 (1.7%)
New NFI by MF or VMT	67 (22.1%)
Other neuritis	23 (7.6%)
**Different types of nerve function impairments (overlapping groups)**	
No sensory NFI	149 (49.2%)
New Sensory NFI	50 (16.5%)
Old sensory NFI	104 (34.3%)
No motor NFI	244 (80.5%)
New motor NFI	33(10.9%)
Old motor NFI	26 (8.6%)
**Old and new NFI by monofilament (overlapping groups)**	
Old sensory loss with no new sensory loss	104 (34.3%)
New sensory loss with or without old sensory loss	50 (16.5%)
Old motor loss with no new motor loss	26 (8.6%)
New motor loss with or without old motor loss	33 (10.9%)
Only old loss, sensory or motor	102 (33.7%)

### Antibody and Cytokine levels in the different Ridley-Jopling classification groups

PGL IgG and IgM antibody levels increased significantly across the Ridley-Jopling spectrum groups with lowest levels in the BT patients and the highest in LL patients (Table 2) (p<0.001). Levels of LAM IgG1 and IgG3 were also significantly higher in BL than BT patients (p<0.01). S100 levels increased across the Ridley-Jopling spectrum with LL patients having higher levels than BT patients (p<0.01). TNFα levels were highly variable with a tendency to be lower in LL patients than patients with BT and BL; however, because of the wide variation, none of the apparent differences reached statistical significance. Antibody and cytokine levels in control sera collected from normal subjects were also tested. Levels of all the antibodies and TNFα were significantly low in controls compared to patients. As shown in Table 2, patients were also classified by bacterial load. PGL IgG and IgM levels were significantly higher (p<0.001) in patients with higher bacterial loads, as were LAM levels in patients with higher bacterial loads. PGL IgG and IgM levels were also significantly higher in smear negative BL compared with smear negative BT patients (p<0.001) (data not shown). S100 levels were also significantly higher in patients with higher bacterial loads.

**Table 2:** Antibody and cytokine mean, standard deviation and median within Ridley Jopling groups and BI groups.

TestMedians and standard devetions	Normal Controls	Leprosy(n = 303)	BT Group(n = 185)	BL Group(n = 89)	LL Group(n = 29)	BI Negative(n = 193)	BI 0.1–3.0(n = 68)	BI 3.1–6(n = 42)
**PGL – IgG** **(AU)**	18.6±18.2(n = 160)	97.7±125.067.0	54.3±38.551.0	147.3±159.8106.0	222.1±205.4162.0	55.5±43.849.0	113.3±72.6104.5	266.5±243.9157.5
**PGL – IgM** **(AU)**	17.9±14.3(n = 160)	153.5±323.971.0	61.4±44.251.0	260.9±471.5106.0	411.1±508.6177.0	73.7±97.154.0	133.6±112.8102.5	552.0±717.5171.0
**LAM – IgG1** **(AU)**	0.5±1.9(n = 160)	11.0±21.22.0	8.9±20.61.0	15.6±23.47.0	10.1±15.63.0	9.6±20.71.0	15.8±25.44.0	10.0±14.74.5
**LAM – IgG3** **(AU)**	1.4±2.5(n = 160)	9.6±16.85.0	7.4±10.54.0	14.3±25.77.0	9.5±12.75.0	7.9±11.44.0	14.3±28.36.0	10.0±11.36.0
**S-100 Antibody** **(AU)**	28.7±28.5(n = 160)	61.9±45.750.0	55.0±38.745.0	71.4±56.055.0	76.8±45.474.0	55.7±39.747.0	62.3±35.259.0	90.0±70.764.5
**Anticeramide Antibody** **(O.D)**	0.2±0.2(n = 286)	0.7±0.50.6	0.7±0.40.6	0.7±0.50.6	0.9±0.70.7	0.7±0.40.6	0.7±0.50.5	0.9±0.70.7
**TNF alpha pg/ml** **(Concentration)**	14.07±45.08(n = 243)	49.0±135.82.3	52.6±138.52.2	55.4±149.82.9	6.0±9.43.1	55.4±141.62.2	38.0±106.62.5	37.0±151.13.7

Notes:

Kruskal-Wallis non-parametric testing used for all significance testing. Baseline serum was available from all 303 cases in the study. Anticeramide was not assessed for 7 individuals. Differences in markers across three Ridley-Jopling groupings reached statistical significance in five markers.

For PGL1_IgG, BT versus BL (p<0.001), BTvLL (p<0.001), BLvLL (p<0.05). Three pairwise comparisons between smear groups all had p<0.001.

For PGL1_IgM, BT versus BL (p<0.001), BTvLL (p<0.001), BLvLL (NS). Three pairwise comparisons between smear groups all had p<0.001.

For LAM_IgG1, BT versus BL (p<0.001), BTvLL (NS), BLvLL (NS). Only Negative smear 1-3 smear reached statistical significance (p<0.01).

For LAM_IgG3, BT versus BL (p<0.001), BTvLL (NS), BLvLL (NS). negative smear versus 1-3 smear (p<0.001) and negative smear versus 3+ smear (p<0.05) reached statistical significance.

For S100, BT versus BL (p<0.01), BTvLL (p<0.05), BLvLL (NS). Only negative smear versus 1-3 smear reached statistical significance (p<0.01).

For Anticeramide, only negative smear versus 3+ smear approached significance (p = 0.054).

### Antibody and Cytokine Levels in reactions

In Table 3 the antibody and cytokine marker levels for patients with and without reactions and NFI are compared. PGL-1 IgG and IgM levels tended to be raised in the presence of nerve damage or reactions but the differences did not reach statistical significance. The lowest mean levels of all seven markers were found when T1R occurred only in the skin with no NFI. A significant decrease in the levels of anti-ceramide antibody levels also occurred when there were T1R with and without new NFI (p<0.01). This might be indicative of a marker that is altered when only skin pathology is occurring. TNF levels were highest in patients with NFI but these differences were not significant. We found no statistically significant association between Ridley-Jopling classification and reaction type.

**Table 3:** Antibody and cytokine mean, standard deviation and median in patients with and without reactions.

Test ValueMean and standard deviation	Patient Groups				
	No reaction(n = 188)	Reaction(n = 115)	Only skin reaction(n = 28)	Skin reaction + NFI(n = 17)	Only NFI(n = 48)
**PGL – IgG** **(AU)**	93.9±120.363.5	103.9±132.674.0	82.6±99.154.0	87.6±69.075.0	102.1±137.073.5
**PGL – IgM** **(AU)**	138.3±265.566.5	178.3±401.673.0	98.9±134.563.0	207.4±439.478.0	175.4±474.271.5
**LAM – IgG1** **(AU)**	11.4±21.61.0	10.4±20.72.0	9.0±28.10.0	11.2±15.3.0	11.4±18.03.5
**LAM – IgG3** **(AU)**	9.5±19.34.0	9.8±11.85.0	8.1±7.94.0	11.2±16.45.0	9.2±9.96.0
**S-100 Antibody** **(AU)**	64.8±48.053.0	57.3±41.647.0	52.1±42.939.0	53.8±28.445.0	56.8±39.647.0
**Anticeramide Antibody** **(O.D)**	0.7±0.50.6	0.7±0.50.6	0.5±0.30.4	0.5±0.30.4	0.8±0.50.7
**TNF alpha** **(Concentration)**	43.9±112.92.1	57.2±166.72.9	13.9±31.92.1	61.9±157.33.2	103.4±232.12.4

Notes:

AU – Arbitrary Units, O.D – Optical Density, Kruskal-Wallis non-parametric testing used for all significance testing. Baseline serum was available from all 303 cases in the study. Anticeramide was not assessed for 7 individuals. For LAM_IgG1, Skin Only versus NFI Only p<0.05, Skin Only versus Skin+NFI p<0.05, For Anticeramide, Skin Only versus NFI Only p<0.01, NFI Only versus Skin+NFI p<0.05.

We used a regression-based approach to assess the more complex relationships between reaction type, Ridley-Jopling type and each cytokine or marker as outcome. Differences achieving statistical significance were as follows:

PGL1_IgG: Differences between BL and BT groups approached statistical significance (p = 0.054). After adjusting for Ridley-Jopling type differences between reaction types failed to reach statistical significance. We found a statistically significant interaction effect (p<0.001), seen in high levels of PGL1_IgM among BL cases with reactions including NFI. S110: We found a statistically significant interaction effect, with LL cases having the lowest levels of S100 and individuals with NFI-only reactions having the highest (p<0.05). TNFalpha: After adjusting for Ridley-Jopling type we found a difference between reactions with both skin and NFI compared with skin-only reactions (p<0.001). BL cases had the highest levels of TNFalpha, particularly among cases with NFI-only reactions (p<0.05).

### Antibody and Cytokine Levels and Nerve Function Impairment

In Table 4 patients were grouped according to the type of NFI that they had. Patients with old sensory NFI had significantly increased levels of PGL IgG, LAM IgG1 and S100 antibody (p<0.05 or less). There were far more patients with sensory NFI (154) compared to motor NFI (59). It was observed that 136 patients did not have any NFI at the time of recuitment. There were 149 patients with no sensory NFI. But out of these 149 patients, 13 patients were with motor NFI (old/new). Similarly there were 244 patients with no motor NFI, out of which 108 patients had sensory NFI (old/new). Patients with no old or new sensory loss. None of the markers were significantly elevated in patients with old and new motor NFI. TNFα levels were non-significantly elevated in patients in both sensory NFI and new motor NFI. An analysis of antibody levels in the 192 smear negative group patients showed that patients with new sensory nerve damage had significantly higher PGL-1 IgG and LAM IgG1 levels (p<0.01 and p = 0.01, respectively) (data not shown). PGL-1 IgM and S100 levels were higher, but the significance level was borderline (p = 0.08 and p = 0.056, respectively). There were no differences between anti-ceramide and TNFα levels in the groups with or without sensory or motor nerve impairment.

**Table 4:** Antibody and cytokine mean, standard deviation and median in patients with different types of nerve function impairment.

	Patient Details								
	No NFI(n = 136)	Sensory NFI				Motor NFI			
		No sensory NFI(n = 149)	New sensory NFI(n = 50)	Old sensory NFI (n = 104)	Only new +/− old sensory NFI (n = 31) (no motor NFI)	No motor NFI (n = 244)	New motor NFI (n = 33)	Old motor NFI (n = 26)	Only new +/− old motor NFI (n = 13) (No sensory NFI)
**PGL – IgG** **(AU)**	79.9±87.959.0	85.3±113.459.0	84.7±53.574.0	122.1±159.173.5	85.5±49.975.0	97.0±117.169.0	109.8±165.466.0	88.6±141.956.0	141.8±260.143.0
**PGL – IgM** **(AU)**	110.8±18963.5	142.3±349.263.0	116.1±126.673.0	188.2±351.972.0	125.8±127.490.0	144.2±254.272.0	241.0±636.462.0	129.8±330.961.5	471.9±986.255.0
**LAM – IgG1** **(AU)**	8.03±18.90.0	7.7±18.20.0	12.6±18.83.5	15.1±25.44.0	10.6±17.71.0	11.2±21.81.0	11.5±17.35.0	9.1±20.50.5	4.4±5.02.0
**LAM – IgG3** **(AU)**	9.4±21.74 .0	8.9±20.84.0	11.2±12.86.0	9.8±11.46.0	11.5±14.86.0	9.9±18.15.0	8.2±8.45.0	8.2±12.54.0	4.4±3.33.0
**S-100 Antibody** **(AU)**	59.2±43.349.0	58.8±42.949.0	52.9±35.445.0	70.9±52.758.0	55.1±39.544.0	61.4±46.449.0	55.2±34.959.0	75.0±50.359.0	54.9±39.139.0
**Anticeramide Antibody** **(OD)**	0.7±0.40.6	0.7±0.50.6	0.6±0.30.6	0.8±0.50.6	0.7±0.40.6	0.7±0.50.6	0.7±0.50.6	0.6±0.60.5	0.8±0.70.6
**TNF alpha** **(concentration)**	39.7±103.12.3	40.1±103.02.3	103.5±238.32.8	35.4±100.22.3	91.1±217.32.2	44.9±124.32.3	96.5±218.23.2	27.0±89.11.0	44.3±105.41.5

Notes:

AU – Arbitrary Units, OD – Optical Density.

Kruskal-Wallis non-parametric testing used for all significance testing.

Baseline serum was available from all 303 cases in the study. Antticeramide was not assessed for 7 individuals.

For PGL1_IgG, No Sensory NFI versus Old NFI p<0.05, (No Sensory NFI versus Old Sensory NFI p = 0.076).

For PGL1_IgM, (No Motor NFI versus Old Motor NFI p = 0.0926).

For LAM_IgG1, No Sensory NFI versus Old NFI p<0.01, No Sensory NFI versus New Sensory NFI p<0.05.

For LAM_IgG3, No Sensory NFI versus Old NFI p<0.01, No Sensory NFI versus New Sensory NFI p<0.01.

For S100, No Sensory NFI versus Old NFI p<0.05, Old Sensory NFI versus New Sensory NFI p<0.01. (No Motor NFI versus Old Motor NFI p = 0.0932).

## Discussion

The INFIR cohort study was a prospective study with carefully designed case definitions and outcomes, which were supported by clinical measurements. The large database has enabled us to analyse the data and correlate the serological findings to specific patient groups. The aim was to detect association between laboratory findings and clinical parameters in a large patient group.

Trends of antibody response in relation to leprosy type and bacterial index were as expected and reported earlier by Schwerer et al [23]. One of the specific objectives was to identify new markers for nerve damage. We found an association between bacterial load, mycobacterial antigens, auto-immunity and inflammation and the development of nerve damage. Our strongest finding was confirming the association between PGL-1 antibody levels and the occurence of reactions and nerve damage. This association is also seen in the association between Ridley-Jopling type leprosy and the presence of reactions or new nerve damage. This association was also present in smear negative patients. This finding has been reported before in a study done in Nepal looking at risk factors for the development of leprosy reactions [8]. It has also been recently reported from a large cohort study in Bangladesh looking for predictive factors for nerve damage [24]. Here the strength of the predictive model was increased from 72% to 80% in the presence of PGL-1 antibodies. One of the limitations for comparison of results with that of normal control sera in this study was that though we used sera from the subjects without neurological disease from the leprosy endemic area, it was not from the same area as that from where the patients were recruited.

We had predicted that S100 and anti-ceramide antibodies would be raised in acute nerve damage but found no evidence of this. However, both antibodies were elevated in patients with old sensory nerve damage. One explanation for this might be that auto-antibodies are not implicated in the initiation of new nerve damage, but once the nerve is damaged, then various nerve antigens may be presented to the immune system, initiating an auto-immune response. This may be one mechanism whereby nerve damage is perpetuated. The ongoing nerve damage seen in treated leprosy patients is an important clinical problem and this association should be investigated further.

Antibodies against LAM were associated with leprosy type, but not with reactions, except with the presence of old sensory NFI. This suggests that LAM might have some role in the initiation of nerve damage, but has little immunogenic role in the ongoing process of nerve damage.

We also found a non-significant association between elevated TNFα levels and new sensory and motor nerve damage. TNFα is a cytokine crucial for granuloma formation and also in the mediation of local tissue damage [25]. TNFα has been reported to cause demyelination and death of oligodendrocytes in a dose dependent manner in *in-vitro* studies [26]. A previous study has demonstrated TNFα in reactional skin and nerve lesions and this finding suggests that TNFα might leak from the nerve lesions into the circulation [19]. A variable elevation of TNFα has been found in ENL reactions [27], [28] and given the close association of TNFα with the pathology of T1R, we predicted that TNFα levels would be elevated. However, the TNF assay detected a wide range of TNFα values. These levels were not easily explicable either in terms of the patients' leprosy pathology or other possible ongoing pathologies. Other reports also document a range of TNF values and a lack of correlation with clinical outcome in leprosy patients [29]. Maybe this is an inherent problem of studying this cytokine in the circulation. We had expected that, with a large number of patients, a tighter range of values might be found. Skin and nerve are very different compartments and it may be that in the context of the leprosy damaged peripheral nerve TNFα leaks more easily into the circulation than from inflamed skin lesions. Andersson et al has shown that in T1R there is marked compartmentalisation of pathology and that cytokines may not leave the skin site for the circulation [29]. This finding will be further tested when the longitudinal data on patients in this cohort who developed reactions and nerve damage during follow up are analysed.

One explanation for the absence of an association between serological markers and motor damage might be that sensory nerve damage is readily detected with monofilaments, whereas motor nerve loss has to be quite advanced before it is detected by the voluntary muscle testing as was used here. In further analyses of the data, more sensitive tests of nerve function such as nerve conduction studies will be analysed and associations between mild motor damage with antibody levels can then be tested [30].

This study did not find a new serological marker for the detection of leprosy reactions. However, a pattern of associations between markers and nerve damage has been shown. These would be consistent with a model of nerve damage which is initiated by mycobacterial antigens such as PGL-1, is maintained by ongoing inflammation through cytokines such as TNFα and then perhaps extended by auto-antibody-mediated nerve damage. It is important that further work should be done to identify markers associated with these different aspects of nerve damage. A small study from Brazil showed that plasma levels of CXCL10 (Inflammatory protein 10 ) and IL6 were raised in 10 patients with T1R [31]. It would be useful to test these marker and other future markers in this well-characterised cohort to evaluate their role in the diagnosis of leprosy reactions.

There are several implications arising from this work. The association of nerve damage with a marker for bacterial load emphasises the need to detect and treat patients as early as possible. However, we also need a marker for nerve damage in patients who are bacteriologically negative, since this study shows that there is also substantial nerve damage occurring in patients who are slit skin smear negative.

## Supporting Information

Checklist S1 STROBE Checklist
(0.10 MB DOC)
