# Supplementary material for: Analysis of Antibody and Cytokine Markers for Leprosy Nerve Damage and Reactions in the INFIR Cohort in India
Source: PLoS Negl Trop Dis. 2011 Mar 8;5(3):e977. doi: 10.1371/journal.pntd.0000977 (PMC3050910; doi:10.1371/journal.pntd.0000977)
Supplement: Checklist S1 — STROBE Checklist (0.10 MB DOC) [file pntd.0000977.s001.doc]

STROBE Statement—checklist of items that should be included in reports of observational studies

|  | Item No | Recommendation |
| --- | --- | --- |
| **Title and abstract** | 1 | (*a*) Indicate the study’s design with a commonly used term in the title or the abstract  Yes. Page 2 |
|  |  | (*b*) Provide in the abstract an informative and balanced summary of what was done and what was found  Yes. Page 2 |
| Introduction | | |
| Background/rationale | 2 | Explain the scientific background and rationale for the investigation being reported  Yes. Page 3-5 |
| Objectives | 3 | State specific objectives, including any prespecified hypotheses  Yes. Page 5,6 |
| Methods | | |
| Study design | 4 | Present key elements of study design early in the paper  Yes. Page 6 |
| Setting | 5 | Describe the setting, locations, and relevant dates, including periods of recruitment, exposure, follow-up, and data collection  Yes. Page 6 |
| Participants | 6 | (*a*) *Cohort study*—Give the eligibility criteria, and the sources and methods of selection of participants. Describe methods of follow-up  Yes. Page 6. The study design was cohort study but the data presented is only of the baseline i.e. at the time of recruitment of the patients.  *Case-control study*—Give the eligibility criteria, and the sources and methods of case ascertainment and control selection. Give the rationale for the choice of cases and controls  *Cross-sectional study*—Give the eligibility criteria, and the sources and methods of selection of participants |
|  |  | (*b*) *Cohort study*—For matched studies, give matching criteria and number of exposed and unexposed  No  *Case-control study*—For matched studies, give matching criteria and the number of controls per case  No |
| Variables | 7 | Clearly define all outcomes, exposures, predictors, potential confounders, and effect modifiers. Give diagnostic criteria, if applicable  Yes. Page 7 |
| Data sources/ measurement | 8* | For each variable of interest, give sources of data and details of methods of assessment (measurement). Describe comparability of assessment methods if there is more than one group  Yes. Page 8-10 |
| Bias | 9 | Describe any efforts to address potential sources of bias  No |
| Study size | 10 | Explain how the study size was arrived at  No. (Described elsewhere) |
| Quantitative variables | 11 | Explain how quantitative variables were handled in the analyses. If applicable, describe which groupings were chosen and why  Yes. Page 8-10 |
| Statistical methods | 12 | (*a*) Describe all statistical methods, including those used to control for confounding  Yes. Page 10 |
|  |  | (*b*) Describe any methods used to examine subgroups and interactions  Yes. Page 10 |
|  |  | (*c*) Explain how missing data were addressed  No |
|  |  | (*d*) *Cohort study*—If applicable, explain how loss to follow-up was addressed  No  *Case-control study*—If applicable, explain how matching of cases and controls was addressed  No  *Cross-sectional study*—If applicable, describe analytical methods taking account of sampling strategy  No |
|  |  | (*e*) Describe any sensitivity analyses  No |

Continued on next page

| Results | | |
| --- | --- | --- |
| Participants | 13* | (a) Report numbers of individuals at each stage of study—eg numbers potentially eligible, examined for eligibility, confirmed eligible, included in the study, completing follow-up, and analysed  Yes. Page 10 |
|  |  | (b) Give reasons for non-participation at each stage  No |
|  |  | (c) Consider use of a flow diagram  No |
| Descriptive data | 14* | (a) Give characteristics of study participants (eg demographic, clinical, social) and information on exposures and potential confounders  No. |
|  |  | (b) Indicate number of participants with missing data for each variable of interest  No |
|  |  | (c) *Cohort study*—Summarise follow-up time (eg, average and total amount)  No |
| Outcome data | 15* | *Cohort study*—Report numbers of outcome events or summary measures over time  Yes. Page 10-12 |
|  |  | *Case-control study—*Report numbers in each exposure category, or summary measures of exposure  No |
|  |  | *Cross-sectional study—*Report numbers of outcome events or summary measures  No |
| Main results | 16 | (*a*) Give unadjusted estimates and, if applicable, confounder-adjusted estimates and their precision (eg, 95% confidence interval). Make clear which confounders were adjusted for and why they were included  No |
|  |  | (*b*) Report category boundaries when continuous variables were categorized  No |
|  |  | (*c*) If relevant, consider translating estimates of relative risk into absolute risk for a meaningful time period |
| Other analyses | 17 | Report other analyses done—eg analyses of subgroups and interactions, and sensitivity analyses  No |
| Discussion | | |
| Key results | 18 | Summarise key results with reference to study objectives  Yes. Page 12-15 |
| Limitations | 19 | Discuss limitations of the study, taking into account sources of potential bias or imprecision. Discuss both direction and magnitude of any potential bias  Yes. Page 13. |
| Interpretation | 20 | Give a cautious overall interpretation of results considering objectives, limitations, multiplicity of analyses, results from similar studies, and other relevant evidence  Yes. Page 12-15 |
| Generalisability | 21 | Discuss the generalisability (external validity) of the study results  Yes. Page 12-15 |
| Other information | | |
| Funding | 22 | Give the source of funding and the role of the funders for the present study and, if applicable, for the original study on which the present article is based  Yes. Page 15 |

*Give information separately for cases and controls in case-control studies and, if applicable, for exposed and unexposed groups in cohort and cross-sectional studies.

**Note:** An Explanation and Elaboration article discusses each checklist item and gives methodological background and published examples of transparent reporting. The STROBE checklist is best used in conjunction with this article (freely available on the Web sites of PLoS Medicine at http://www.plosmedicine.org/, Annals of Internal Medicine at http://www.annals.org/, and Epidemiology at http://www.epidem.com/). Information on the STROBE Initiative is available at www.strobe-statement.org.
